# Microsurgical Salvage of Neonatal Upper Limb Ischaemia Following Intrauterine Brachial Vessel Constriction

**DOI:** 10.7759/cureus.29777

**Published:** 2022-09-30

**Authors:** Jamie Clements, Harry Lewis, Michael McBride

**Affiliations:** 1 Plastic Surgery, Ulster Hospital, Belfast, GBR; 2 Plastic and Reconstructive Surgery, Ulster Hospital, Belfast, GBR

**Keywords:** congenital birth defect, acute limb ischaemia, supermicrosurgery, limb salvage procedures, microsurgery

## Abstract

Neonatal limb ischaemia is a rare disease entity with devastating morbidity, including compartment syndrome, tissue loss, limb loss, reduced limb growth, irreparable neuropathies and Volkmann’s syndrome.

We report a case of limb revascularisation and salvage due to intrauterine brachial artery thrombosis. Published literature is limited to case reports and case series, with various treatment modalities discussed. Early recognition, prompt institution of appropriate treatment and monitoring is vital to achieve successful revascularisation and prevention of life-long morbidity.

A male baby at (36+6week) gestation was born to a nulliparous mother with gestational diabetes via uncomplicated elective caesarean section. Aetiology was due to dense fibrotic circumferential constriction of the brachial vessels and plexus. Successful revascularization was achieved with a contralateral interposition reversed great saphenous vein graft.

Though extremely rare and the clinical presentation varies with the location and timing after birth, the surgeon should maintain a low threshold for suspicion of in the presence of the characteristic sequelae of ischaemia. Doppler ultrasonography can aid the diagnosis where ambiguous, and therapy should be individualised based on the clinical presentation; this case emphasises the role of surgery in limb salvage.

## Introduction

We report a case of limb revascularisation and salvage due to intrauterine brachial artery compression. Neonatal limb ischaemia is a rare disease entity with devastating morbidity and mortality. The newborn haemostatic pathways and both intrauterine and postnatal events are felt to be precipitating factors. Cases have been reported across the literature, with various treatment modalities discussed - medical, endovascular and surgical. Consensus varies regarding management. Early recognition, prompt institution of appropriate treatment and monitoring is vital to achieve successful revascularisation and prevention of life-long morbidity.

This article was previously presented as a poster at the ASIT x MedAll Surgical Summit on 17th October 2020.

## Case presentation

A full-term (36+6week) male baby was born to a nulliparous mother via elective caesarean section. Her antenatal history was notable for controlled gestational diabetes mellitus. There were no other pre- or peri-natal issues. All antenatal scans were normal. Family history was unremarkable - no thrombotic or ischaemic diseases. 

Delivery was uncomplicated. The umbilical cord and placental gross examination were normal. Birth weight was appropriate, and Apgar scores were normal. 

**Figure 1:**
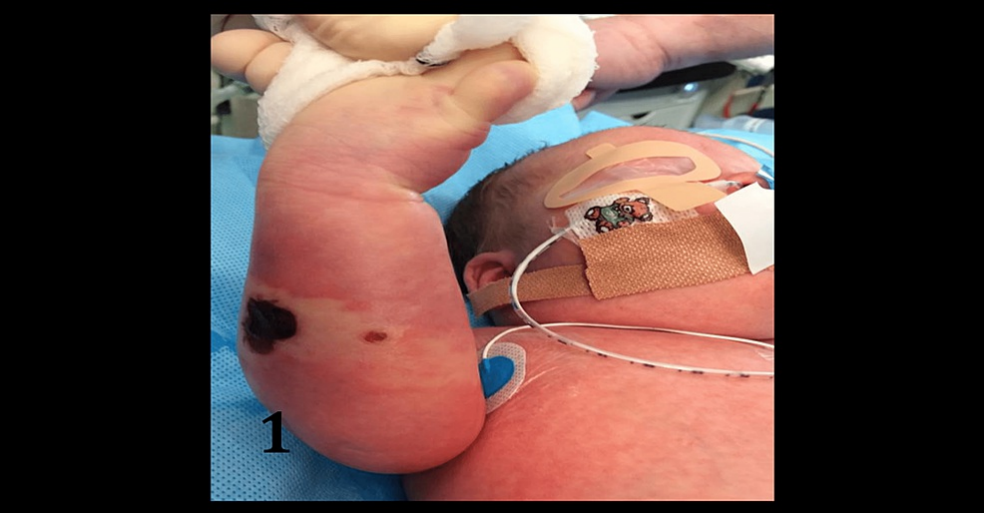
Ischaemic compromise of limb evidenced by the focus of necrosis with surrounding swelling and pallor

Post delivery, the patient was noted to have an oedematous, ecchymotic right forearm and hand. - With small foci of epidermolysis and dry necrosis on the dorsal forearm (Figure 1).

The limb was cool and hypovascular, with a capillary refill time of five to six seconds. Brachial, radial, and ulnar pulses could not be felt clinically or detected sonographically. The volar forearm compartment was tense, but the remaining compartments were normal. The limb demonstrated no spontaneous motor movement - asymmetric moro and grasp reflexes. Limb assessment elicited patient distress indicating no sensory deficit. 

The remaining examination demonstrated an innocent systolic murmur (grade 1/6). Bedside transthoracic echocardiography was normal. Additional examination was normal, including growth parameters.

A plain radiograph of the chest and upper limb showed no cervical rib fracture or dislocation. Doppler ultrasound of the right upper limb showed no arterial flow from the upper 1/3 brachial artery distally - no additional findings were reported. 

Blood parameters indicated a normal complete blood count, C-reactive protein, erythrocyte sedimentation rate (ESR), renal profile, liver profile, coagulation, vasculitic and thrombophilia screen. Creatine kinase (CK) measured 7500 U/L (reference values age <3 months not established, but >3 months 39-308 U/L).

**Figure 2:**
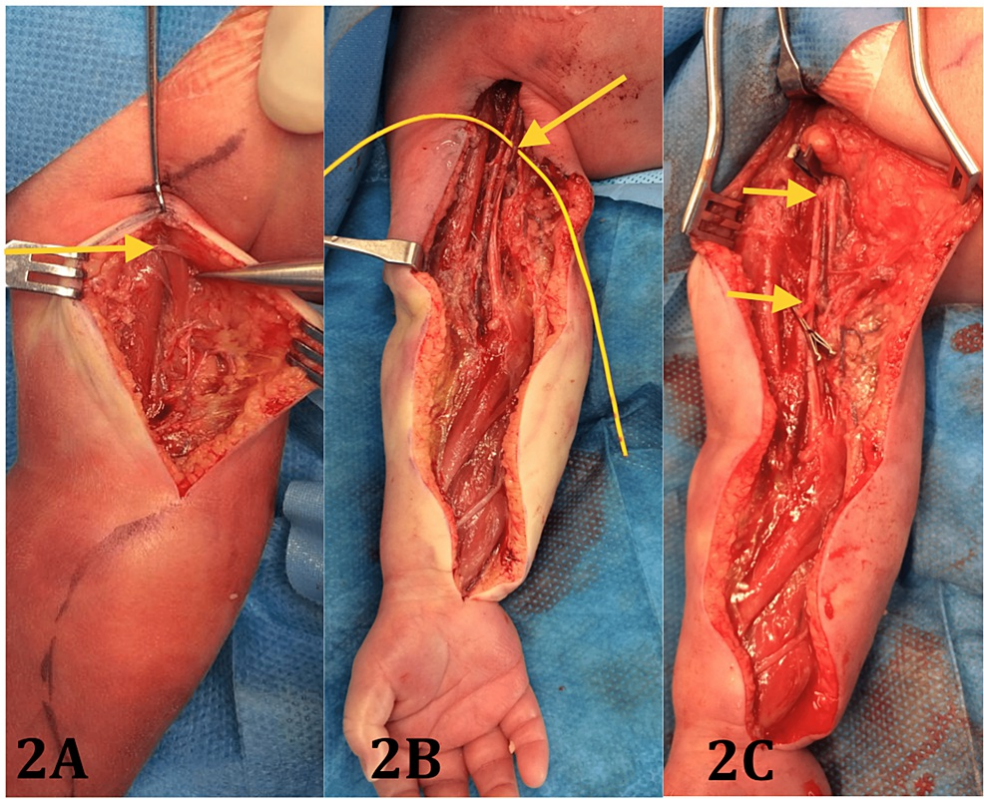
An anomalous ‘Langer’s’ axillary arch demonstrated at the forcep tip (2A), with proximal venous engorgement of slooped axillary vein (2B), and arterial defect between microsurgical clamps following vessel preparation (2C)

Initial management was with topical nitrates, but this was ineffective. The patient proceeded to the emergency exploration of the right arm/ forearm under general anaesthetic on day two. Findings included an anomalous Langer’s axillary arch, dense fibrotic circumferential constriction of the brachial vessels and plexus, with proximal venous engorgement and distal intraluminal brachial artery thrombosis over a 3cm segment, including the profunda brachii origin (Figure 2).

An anomalous arterial branch was noted, arising directly off the brachial artery cephalic to the pathological constricted segment supplying collateral flow to the limb. 

**Figure 3:**
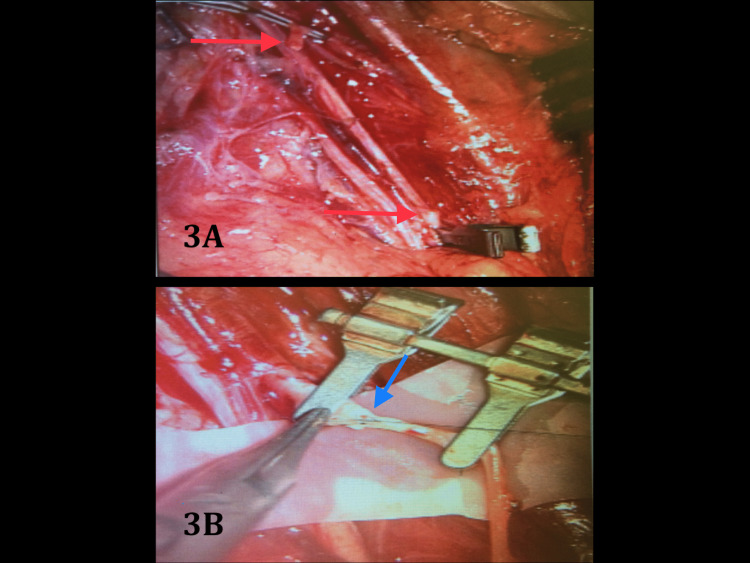
Arterial defect demonstrated between microsurgical clamps and red arrows (3A), with interposition reverse great saphenous vein graft inset below (blue arrow; 3B)

The thrombosed brachial artery segment was excised and reconstructed with a contralateral interposition reversed great saphenous vein graft. Vessel calibres were 3 mm (brachial artery) and 0.5 mm (great saphenous vein), respectively (Figure 3).

**Figure 4:**
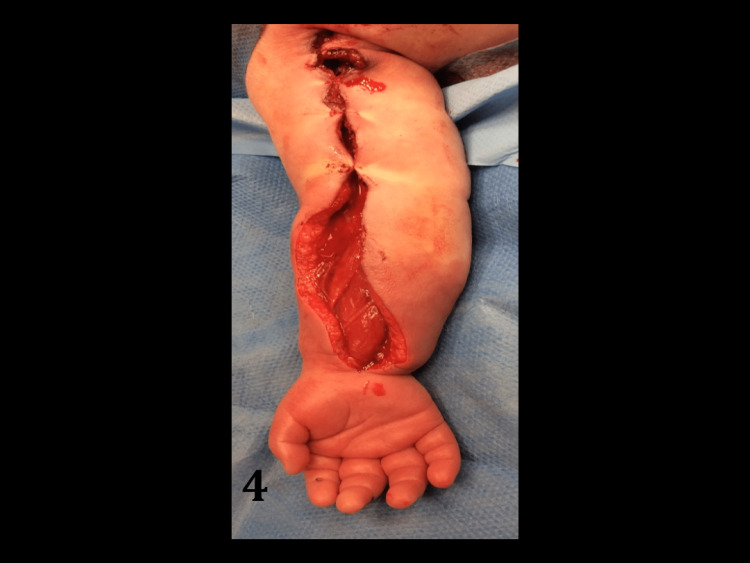
Post-operative limb appearances demonstrating improved cutaneous blood flow

The profunda brachii was not reconstructed. The volar forearm compartment was fasciotomised - muscle appearing viable and improved following successful limb revascularisation (Figure 4).

The patient was transferred to the intensive care unit for sedation, ventilation and monitoring - no additional organ support was required. Systemic heparinisation was instituted for 24 hours, with daily low molecular weight heparin for. All biochemical indices were normalised with supportive therapy. He underwent delayed split-thickness skin graft reconstruction to the volar fasciotomy wound. At the six month follow-up, the limb demonstrated normal vascularity and normal sensorimotor function (Medical Research Council (MRC) grade 5 power globally).

## Discussion

Neonatal limb ischaemia is a rare disease entity, with the incidence of symptomatic neonatal thromboembolism reported to be 0.5 per 10,000 live births [1]. The potential morbidity can be devastating, including compartment syndrome, tissue loss, limb loss, reduced limb growth, irreparable neuropathies and Volkmann’s syndrome, all with life-long sequelae.

Neonatal compartment syndrome with impending Volkmann’s contracture has been reported throughout the literature. Armstrong et al. reported six cases over 12 years - the majority were managed conservatively. Ragland et al. reported on their experience of 24 cases of idiopathic unilateral forearm compartment syndrome in newborns over a 20-year period. Notably, all cases had a similar clinical presentation to our patient - soft tissue skin lesion. No obvious aetiologies were noted, and there were no patterns of risk factors in this cohort. Only one patient was evaluated and treated by a hand surgeon during the first 24 hours, with normal hand function at the one year follow-up. The remaining cases were assessed late (between the ages of 0-13), with established Volkmann’s ischaemic contracture with variable muscle, bone and neural morbidity. They all required multiple staged reconstructive procedures with variable long-term function [2,3].

Neonates have different concentrations and availability of coagulation and fibrinolytic components when compared with older children and adults. The concentrations of clotting factors, thrombin production and coagulation inhibitors are lower, and they are therefore at increased risk of both bleeding and thrombotic complications [4].

Precipitating events causing neonatal limb thromboembolism and /or ischaemia may be intrauterine or post-natal. Greater than 90% of thromboembolic cases in neonates are post-natal and associated with iatrogenic events - arterial and/or venous access devices. Additional risk factors include maternal lupus, maternal diabetes, birth asphyxia, neonatal polycythaemia, sepsis, poor cardiac output, and dehydration [5].

Rarely the thromboembolic event occurs in the intrauterine period. The exact pathological mechanism is unknown. Both extraluminal and intraluminal mechanisms have been proposed as risk factors. Extrinsic causes range from mechanical compression to fetal posture and oligohydramnios, umbilical cord loops, amniotic band construction, and direct birth trauma. Intraluminal theories reference the abnormal haemostatic pathways discussed earlier [3].

Clinical presentation varies with the location and timing after birth. The initial diagnosis is based on the characteristic sequelae of ischaemia. Doppler ultrasonography can aid the diagnosis, with angiography useful in certain cases for pre-planning and/or following treatment. 

The published literature is limited to case reports and case series of various institutions and is subsequent to positive reporting bias. To date, there are no randomised controlled trials comparing different treatment therapies. Authors have published a number of treatment algorithms [5-7].

Therapy should be individualised based on the clinical presentation. First-line treatment consists of unfractionated heparin, with or without thrombolysis. These measures must be instituted with a high degree of caution and vigilance, and if non-surgical modalities are contraindicated, ineffective, or clinical presentation is severe, then surgery should not be delayed. The consensus regarding the criterion, timing and operative approach varies in the literature. 

Surgical options described by multiple authors include decompression of the affected limb to augment the residual vascular flow; open thrombectomy (linear arteriotomy with atraumatic milking of thrombus); and segment arteriectomy with interposition reversed vein graft. The use of a small Fogarty catheter has also been reported to remove embolus, but many authors have reported difficulty with this technique: vessel wall intimal damage or rupture [8,9].

## Conclusions

Neonatal limb ischaemia is a rare disease entity with devastating morbidity- including compartment syndrome, tissue loss, limb loss, reduced limb growth, irreparable neuropathies and Volkmann’s syndrome. The newborn haemostatic pathways and both intrauterine and postnatal events may be precipitating factors. Clinical presentation varies with the location and timing after birth. The initial diagnosis is based on the characteristic sequelae of ischaemia. Doppler ultrasonography can aid the diagnosis. Therapy should be individualised based on the clinical presentation. With advances in neonatal anaesthesia and, notably, the evolution of microsurgery, surgical treatment is now a safe and effective option. Early recognition, prompt institution of appropriate treatment and monitoring is vital to achieve successful revascularisation and prevention of life-long morbidity.
